# Jointly Learning Multiple Sequential Dynamics for Human Action Recognition

**DOI:** 10.1371/journal.pone.0130884

**Published:** 2015-07-06

**Authors:** An-An Liu, Yu-Ting Su, Wei-Zhi Nie, Zhao-Xuan Yang

**Affiliations:** School of Electronic Information Engineering, Tianjin University, Tianjin 300072, China; Nanjing University of Aeronautic and Astronautics, CHINA

## Abstract

Discovering visual dynamics during human actions is a challenging task for human action recognition. To deal with this problem, we theoretically propose the multi-task conditional random fields model and explore its application on human action recognition. For visual representation, we propose the part-induced spatiotemporal action unit sequence to represent each action sample with multiple partwise sequential feature subspaces. For model learning, we propose the multi-task conditional random fields (MTCRFs) model to discover the sequence-specific structure and the sequence-shared relationship. Specifically, the multi-chain graph structure and the corresponding probabilistic model are designed to represent the interaction among multiple part-induced action unit sequences. Moreover we propose the model learning and inference methods to discover temporal context within individual action unit sequence and the latent correlation among different body parts. Extensive experiments are implemented to demonstrate the superiority of the proposed method on two popular RGB human action datasets, KTH & TJU, and the depth dataset in MSR Daily Activity 3D.

## Introduction

Human action recognition is an essential issue in computer vision and machine learning due to its wide and significant applications on multimedia content analysis and retrieval, human computer interaction and so on [1]. Recently, the importance is strongly highlighted by the urgent need for intelligent video surveillance in security sensitive environments.

### Motivation and Overview

Although the current methods have shown the superior performance on this task, there still exist two problems.

- Only leveraging the global characteristics of one action while seldomly considering the body part information. One kind of methods utilize the bag-of-visual-word model with space-time interest point detectors and descriptors for global visual representation [2] [3]. The silhouette-based method is another representative method, which has been widely utilized for action recognition [4]. However, this kind of methods highly rely on accurate foreground extractions, which seriously limits their application. Since human action usually shows a dynamic visual pattern, many sophisticated sequential modeling methods have been designed for this task. Grammar-based methods [5, 6] have been successfully applied for complex human activity recognition. Pei *et al*. [7] proposed a Stochastic Context Sensitive Grammar-based method for video event prediction. They constructed the hierarchical structure to represent the spatial and temporal relationships between the sub-events. The syntactic-based methods are another widely used method to represent human activity with high level temporal logic complexity. Hamid *et al*. [8] proposed to encode the sequential dynamic within human activities with a set of local action units. Consequently, they handled the problem of discovering action dynamic by feature selection. Graphical model-based methods [9] have recently attracted great interests due to its strong ability for sequential modeling. Lv *et al*. [10] trained the hidden Markov model with 3D joint trajectories to model the dynamic within human motion. Han *et al*. [11] utilized the conditional random fields model for the identification of continuous human activity. As the extension of the conditional random fields model, multiple advanced graphical models, including the hidden conditional random fields [12], the latent dynamic conditional random fields [13], the bidirectional-integrated random fields model [14], the semi-Markov model [15], etc., were developed for sequential modeling. Although these powerful methods have shown great performances on this task, they only consider the sequential dynamics of entire body within one action. Since the visual features of different body parts might lie in different feature subspaces, it would be difficult to learn the latent state spaces of one action by simply considering the sequential visual features of the entire body. Consequently, it is mandatory to take advantage of the characteristics of the body and parts together.
- Ignoring the sequential dynamics of body part. Recently, much more work has been done on the part-based method since part-based representation and modeling is more discriminative by focusing on local regions [16]. The most famous part-based method is the deformable part models (DPM) [17] which can simultaneously learn the characteristics of local and global body regions and their correlation with the latent support vector machine. Motivated by the DPM method, Wang *et al*. [18] [19] leveraged the motion patterns of both part regions and body region to construct a graphical model with the latent states for human action recognition. To avoid these highly structured models, Sharma *et al*. proposed the expanded parts model to automatically discover the parts and learn corresponding discriminative templates with their respective locations from a pool of candidate pars. Tian *et al*. [20] further improved the 2D DPM method, which used to be utilized for the still image, and designed the 3D DPM method to model the local and global spatiotemporal action pattern. We also propose the part-regularized multi-task structural learning method for both multiple-view and single-view action recognition [21]. This method can couple the body-based classification and the part-based classification to benefit intrinsic relatedness sharing across multiple action categories and consequently augment the performance of action recognition. These methods ignore the temporal context of partwise features for action modeling. Therefore, the specific sequential modeling methods need to be developed to take advantage of the sequential partwise features for temporal dynamic modeling.

To tackle both problems, it is mandatory to develop a method, which can take advantage of both global temporal context and partwise temporal context within one action to discover both sequence-specific structure and the sequence-shared correlation for action modeling. In this paper, we propose the multi-task conditional random fields model and explore its application on the task of human action. First, we partition human body into several parts with the prior knowledge of body structure and propose the partwise spatio-temporal action unit sequence (ST-AUS) to represent the temporal context of individual body part during one action. Second, we propose the multi-task conditional random fields (MTCRFs) model to jointly learn sequential dynamics of entire body and individual parts and the correlation inbetween. We demonstrate the superiority of the proposed method on on two RGB human action datasets, KTH [22] & TJU, and the depth dataset in MSR Daily Activity 3D [23].

### Contributions

The main contributions of the proposed method can be summarized as follows.

- Motivated by the theory of multi-task learning (MTL) [24], we originally propose the multi-task conditional random fields (MTCRFs) model. Different from the direct feature-level fusion and decision-level fusion, the proposed MTCRFs can learn both temporal structure within individual partwise spatio-temporal action unit sequence and transfer the latent correlation inbetween. Therefore, it can preserve the dynamics of individual sequence while sharing the complementary information of different body parts.
- We propose the partwise spatio-temporal action unit sequence (ST-AUS) to represent the multi-level visual dynamics. Individual ST-AUS can represent the part-specific visual dynamic while multiple ST-AUSs together can convey the latent correlations among multiple body parts. Therefore, the proposed ST-AUS representation can well depict the diverse visual characteristics of each action video.
- We contribute a novel human action dataset (TJU) to the community. TJU contains 22 types of human actions. There are totally 1760 action sequence. The synchronized RGB/depth/skeleton sequences of one action were taken with a Microsoft Kinect sensor with 20fps frame rate and 640 × 480 resolution. To obtain the satisfactory skeleton data, the dataset was recorded in the lab and no occlusion was set. However, it is still challenging since there are more complex and similar actions and both light and dark environments are concerned. This dataset can be downloaded from http://dx.doi.org/10.7910/DVN/YDDJ9F.

The rest of the paper is organized as follows. Section 2 briefly introduces the framework of the proposed method. Section 3 and 4 introduce the proposed spatio-temporal action unit sequence and the multi-task conditional random fields model, respectively. Section 5 explains the experimental method and Section 6 illustrates the experimental results. At last, we conclude the paper in Section 7.

## Framework

The proposed method aims to automatically identify the action performing in one query video by discovering and modeling both partwise and bodywise dynamics. It contains two key steps, ST-AUS representation and MTCRFs modeling.

**Fig 1:**
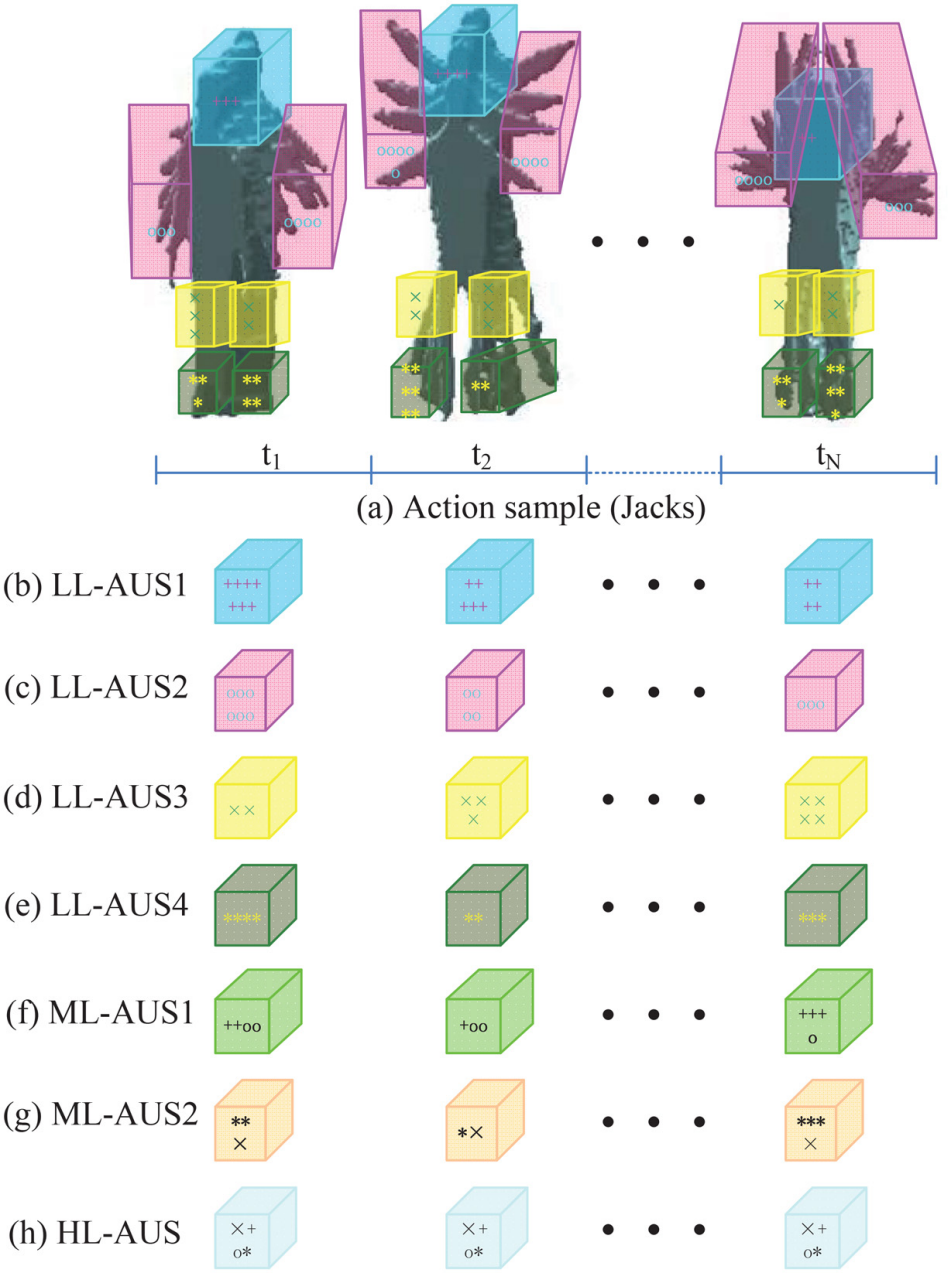
Partwise spatio-temporal action unit sequence. Note that *,×,+,o respectively denote the space-time interest points in different part areas. Different colors denote different body parts.

1) **ST-AUS representation**: We propose the partwise spatio-temporal action unit sequence (ST-AUS) as shown in Fig 1. We utilize the prior knowledge of body structure to define seven body parts, e.g., head, left/right limbs, left/right legs, and left/right feet. One specific part region lasting for T frames is considered as an action unit. Then the action units belonging to one part region in an action video is considered as a partwise action unit sequence. Each partwise action unit sequence focuses on the dynamic of specific body area. Consequently, each action video can be represented in multiple sequential feature spaces.

2) **MTCRFs modeling**: With the ST-AUS representation, we propose the multi-task conditional random fields (MTCRFs) model. The partwise spatio-temporal action unit sequences can be considered as the sequential observations of MTCRFs to discover the latent state space and the transition inbetween. Since different partwise spatio-temporal action unit sequences might have different temporal dynamics, they cannot be linked or fused directly. In MTCRFs, each sequential observation is designed to connect to one sequential hidden state and all the hidden state sequences are correlated to the sequence label. Therefore, the proposed MTCRFs can flexibly learn the temporal dynamics of individual sequence and discover the correlation among them.

## Spatio-temporal Action Unit Sequence

The proposed spatio-temporal action unit sequence(ST-AUS) representation contains three main steps (Fig 1).

First, we extract the local saliency descriptors. The extraction of local space-time feature can be accomplished by local saliency point detection and description. The popular local space-time interest point detectors and descriptors [25] [26] [27] [28] [29] can be implemented for this step. With the extracted local space-time interest points, one video can be considered as a collection of local spatiotemporal points. We will further partition them into different groups depending of the prior knowledge of human body structure.

**Fig 2:**
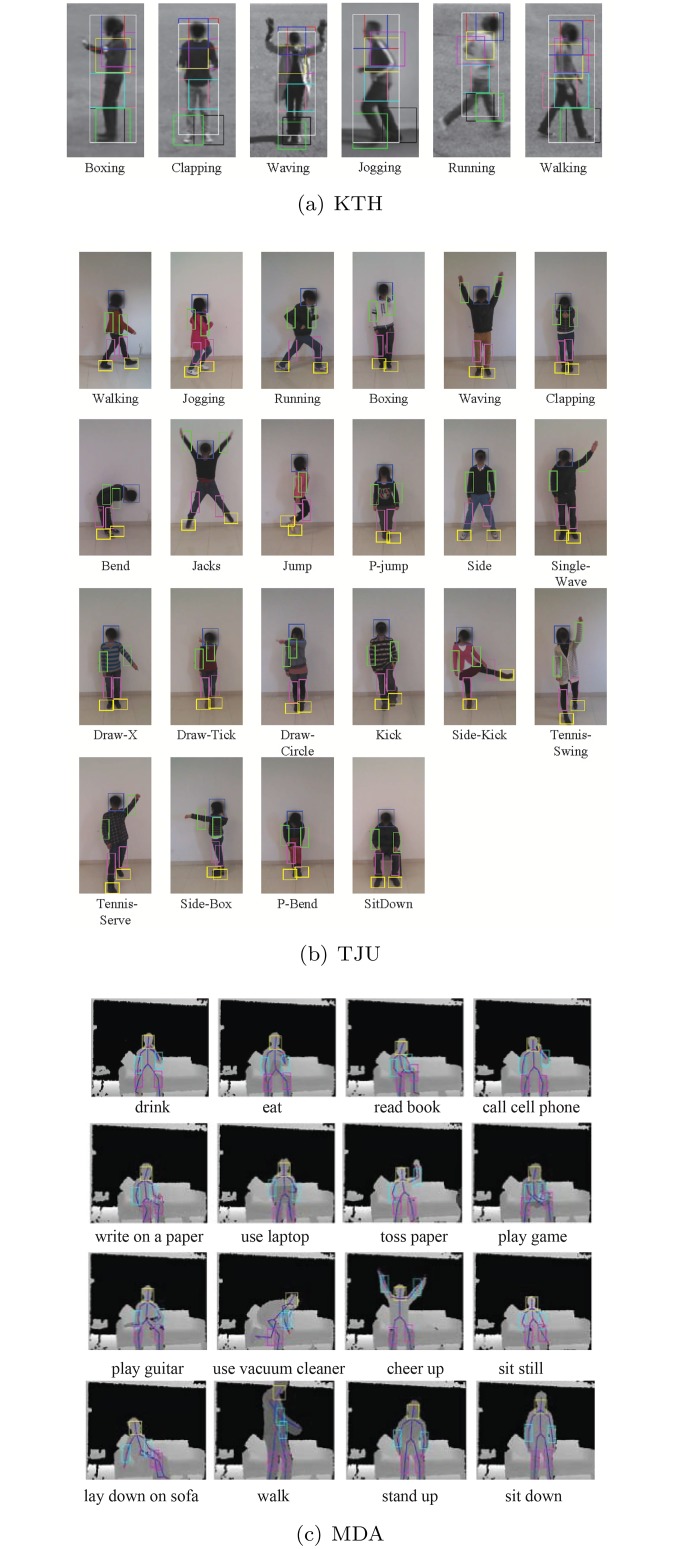
Samples from KTH (a), TJU (b), and MDA (c).

To achieve the body structure information, we implement two methods for body part localization: 1) For the classic RGB human action datasets, we implement the part model-based method [17] to localize 7 body parts (head, left/right limbs, left/right legs, and left/right feet). Fig 2a shows the samples from KTH. In each image, the big box denotes the localization of human body. The 7 small boxes denote the localized part regions. 2) For the recent datasets recorded by the Kinect sensor, the skeleton data can be directly used for body part localization. Fig 2b and 2c show the samples of the skeleton-based localization results on TJU and MDA.

With the localized part regions, we can obtain the centers of individual regions and then utilized them to group the space-time interest points into different part-induced categories. Depending on different feature pooling methods, the proposed ST-AUS can be classified into three levels.

**Low Level (LL) ST-AUS**: LL ST-AUS represents the basic partwise visual dynamics. The left and right limbs/legs/ feet are respectively considered as one category to avoid the sparse interesting points in individual regions. There are totally 4 kinds of parts, including head/limb/leg/foot and consequently the space-time interest points can be grouped into the corresponding categories. Then, we can learn individual codebook with the K-means algorithm. The specific part regions in adjacent *F* frames is defined as a spatio-temporal action unit (ST-AU) which represent the local saliency of a specific body part locating in a special spatio-temporal volume. Therefore the dynamic evolution of a specific body part performing an action can be represented by the sequential ST-AUs, which is defined as the spatio-temporal action unit sequence (ST-AUS). With the learned dictionary corresponding to each part, the standard Bag-of-Words (BoW) scheme can be implemented on each ST-AU for BoW representation and consequently ST-AUS can be represented by the sequential BoW features. Fig 1b-1e show the BoW representations for four different partwise ST-AUS, including Head ST-AUS (LL-AUS1), Limb ST-AUS (LL-AUS2), Leg ST-AUS (LL-AUS3), and Foot ST-AUS (LL-AUS4).

**Middle Level (ML) ST-AUS**: ML ST-AUS represent the composite partwise visual dynamics. The space-time interest points in head/limb regions can be integrated into the same group to represent the upper-body characteristics while the space-time interest points in leg/foot regions can be integrated into the other group to represent the lower-body characteristics. Consequently, we can get the Upper ST-AUS (ML-AUS1) and Lower ST-AUS (ML-AUS2) (Fig 1f-1g), as we do for LL ST-AUS representation.

**High Level (HL) ST-AUS**: HL ST-AUS focuses on the global visual dynamics. The space-time interest points of one person are considered as one group for codebook learning. The feature representation of the entire body, Full ST-AUS (HL-AUS) (Fig 1h), can be generated as we do for LL/ML ST-AUS representation.

LL ST-AUS, ML ST-AUS and HL ST-AUS together form the ST-AUS representation, ST-AUS = {LL-AUS1, LL-AUS2, LL-AUS3, LL-AUS4, ML-AUS1, ML-AUS2, HL-AUS}, for one action video, which conveys different partwise temporal structures.

## Multi-task Conditional Random Fields

In this section, we respectively present the probabilistic model of the multi-task conditional random fields (MTCRFs) model and the methods for MTCRFs learning and inference.

### Probabilistic Model of MTCRFs

**Fig 3:**
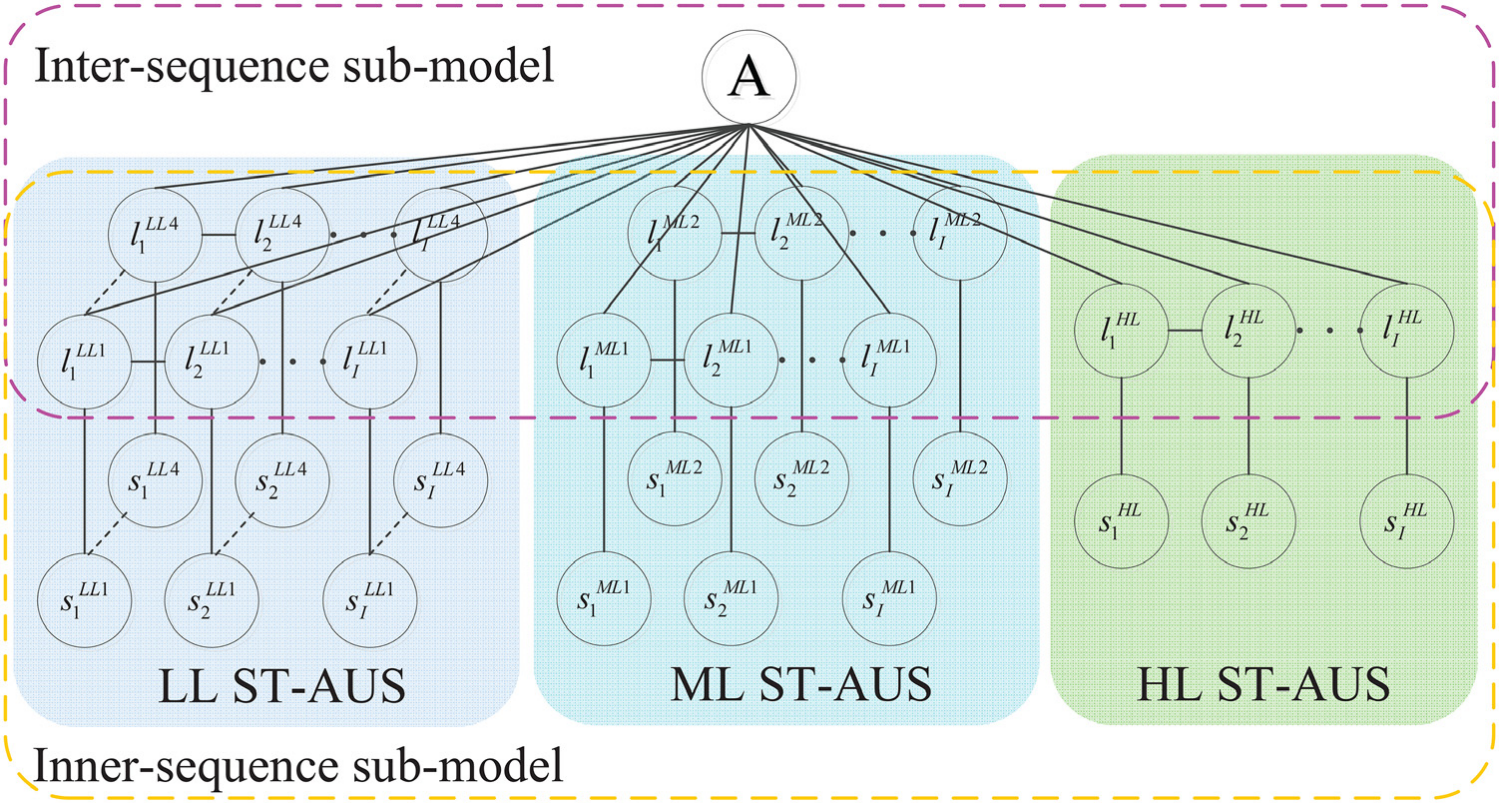
Graph structure of MTCRFs.

We design the specific graph structure 𝓖 = {𝓥, 𝓔} (Fig 3) for the MTCRFs model. 𝓥 means the node set, including both observation nodes and hidden state nodes. ℰ means the edge set, including the transition between adjacent hidden states and the correlation between the hidden state and the action label. In terms of the designed graph structure, each action video, depicted by the extracted ST-AUS representation, can be represented by *P* parallel sequences $S={\left\{s^{p}\right\}}_{p=1}^{P}$ with the chain structure, where $s^{p}={\left\{s_{i}^{p}\right\}}_{i=1}^{I}$ denotes individual part-induced ST-AUS which represents the temporal dynamics of a specific body part during one action. *S* is assigned with a specific action label *A* ∈ 𝓐. To model the state transition within individual ST-AUS (*s* ^*p*^), we utilize the hidden state layer $L={\left\{l^{p}\right\}}_{{}^{{}_{p=1}}}^{P}(L\in 𝓛)$ to correlate the adjacent observations, in which $l^{p}={\left\{l_{i}^{p}\right\}}_{i=1}^{I}$ means the hidden state sequence corresponding to *s* ^*p*^. Each $l_{i}^{p}$ is a member of a finite discrete set 𝓛^*p*^ of the *p* ^*th*^ ST-AUS. All the hidden states are correlated by the edge between individual hidden state and the action label node. Consequently, all ST-AUSs can be correlated and will contribute for the modeling and inference of the action category.

To take advantage of both inner-sequence and inter-sequence context for MTCRFs modeling, the proposed probabilistic model of MTCRFs can be formulated with two parts:

$$\begin{array}{c} P\left(A|S,\Theta \right)=P_{1}\left(A|S,\phi \right)+P_{2}\left(A|S,\psi \right) \end{array}$$ (1)

where Θ = {*ϕ*, *ψ*} denotes the weight coefficients of the model; *P*_1_(*A*∣*S*, *ϕ*) and *P*_2_(*A*∣*S*, *ψ*) denotes the inner-sequence sub-model and the inter-sequence sub-model, respectively.

The inner-sequence sub-model *P*_1_(*A*∣*S*, *ϕ*) can be formulated as follows:

$$\begin{array}{c} P_{1}\left(A|S,\phi \right)=\sum_{L}P\left(A,L|S,\phi \right)=\frac{1}{Z_{1}}\sum_{L}\exp\left(\phi^{⊤}\cdot \mu \left(A,S,L\right)\right) \end{array}$$ (2)

where *Z*_1_ is the partition function for normalization. The potential function of the inner-sequence sub-model, *ϕ* ^⊤^⋅*μ*(*A*, *S*, *L*) ∈ ℝ, can be defined with the first-order attribute between the observation and the corresponding hidden state and the second-order attribute between adjacent hidden states:

$$\begin{array}{c} \begin{array}{cc} \phi^{⊤}\cdot \mu \left(A,S,L\right)= & \sum_{p=1}^{P}\sum_{i=1}^{I}\sum_{k=1}^{K_{1}}\phi_{1,k}F_{1,k}\left(l_{i}^{p},s_{i}^{p}\right)\\ & +\sum_{p=1}^{P}\sum_{i=1}^{I}\sum_{k=1}^{K_{2}}\phi_{2,k}F_{2,k}\left(l_{i}^{p},l_{i+1}^{p}\right) \end{array} \end{array}$$ (3)

where the first term denotes the correlation between the observation $s_{i}^{p}$ and the corresponding hidden state $l_{i}^{p}$ and the second term denotes the transition between adjacent hidden states. $F_{1,k}(l_{i}^{p},s_{i}^{p})\in \mathbb{R}$ is a general expression of the first order attribute function which represents the relationship between pairwise observation and hidden state nodes. $F_{2,k}(l_{i}^{p},l_{i+1}^{p})\in \mathbb{R}$ is a general expression of the second order attribute function which represents the relationship between pairwise hidden state nodes. Different from previous work [30] which only consider the first order attribute between the observation node and the corresponding hidden state node, we leverage both first order and second order attributes to formulate the inner-sequence sub-model. It is intuitive that different actions might have the similar action unit. For example, both boxing and handclapping can have similar hand/limb motion. It is not discriminative enough to distinguish each other only with the body motion during one state. However, it will be more easily to discriminate both when considering the motion changes between adjacent states since there will be different motion intensity, direction, etc. These motion changes can be well represented by the second-order attribute.

The inter-sequence sub-model *P*_2_(*A*∣*S*, *ψ*) can be likewise formulated as follows:

$$\begin{array}{c} P_{2}\left(A|S,\psi \right)=\sum_{L}P\left(A,L|S,\psi \right)=\frac{1}{Z_{2}}\sum_{L}\exp\left(\psi^{⊤}\cdot \nu \left(A,S,L\right)\right) \end{array}$$ (4)

where *Z*_2_ is another partition function for normalization. The potential function of the inter-sequence sub-model, *ψ* ^⊤^⋅*ν*(*A*, *S*, *L*) ∈ ℝ, can be defined with the first-order attribute between the sequence class and the corresponding hidden state and the second-order attribute between the sequence class and the pairwise hidden states:

$$\begin{array}{l} \psi^{⊤}\cdot \nu (A,S,L)=\sum_{p=1}^{P}\sum_{i=1}^{I}\sum_{A^{\prime }\in A}\sum_{k=1}^{K_{3}}\psi_{1,k}{F^{\prime }}_{1,k}(l_{i}^{p},A^{\prime })\\ \;\;\;\;\;\;\;\;\;\;\;\;\;\;\;\;\;\;\;\;\;\;\;\;\;\;+\sum_{p=1}^{P}\sum_{i=1}^{I}\sum_{A^{\prime }\in A}\sum_{k=1}^{K_{4}}\psi_{2,k}{F^{\prime }}_{2,k}(l_{i}^{p},l_{i+1}^{p},A^{\prime }) \end{array}$$ (5)

where the first term denotes the correlation between the sequence label *A* and individual hidden state $l_{i}^{p}$ and the second term denotes the correlation between the sequence label *A* and pairwise hidden states. $F_{{}_{1,k}}^{\prime }(l_{i}^{p},A^{\prime })\in \mathbb{R}$ is the first order attribute function which represents the relationship between the sequence label and individual hidden state node. $F_{{}_{2,k}}^{\prime }(l_{i}^{p},l_{i+1}^{p},A^{\prime })\in \mathbb{R}$ is the second order attribute function which represents the relationship between the sequence label and pairwise hidden state nodes. Since all the hidden states are linked by the sequence label node, this model can learn the latent correlation among different ST-AUSs. This is quite understandable since each action has specific motion pattern and the movement of different body parts during one action is implicitly constrained by each other. Different from previous work [31] which designed the edges between the pairwise hidden states from two different chain-structured sequences to learn the inter-sequence correlation, we omit this edges due to two reasons: 1) there might exist asynchrony among different ST-AUSs since different part-induced ST-AUSs can have different motion dynamics. It is unreasonable to impose strong constraint by linking the pairwise hidden stated with the same index from two sequences. 2) the inter-sequence edges linking pairwise hidden states will significantly increase the complexity of the graph structure and directly make model learning much more difficult.

### MTCRFs Learning and Inference

Since both sub-models have the similar formulation in the exponential family manner, they can be separately optimized by the gradient descent method [32]. Here we take the inner-sequence sub-model, *P*_1_(*A*∣*S*, *ϕ*), as an example to illustrate the model learning method. Model learning of *P*_1_(*A*∣*S*, *ϕ*) can be accomplished by maximize the following likelihood objective function, given the training set with N samples $\Gamma ={\left\{\left(S^{i},A^{i}\right)\right\}}_{i=1}^{N}$:

$$\begin{array}{c} L\left(\Gamma ;\phi \right)=\sum_{(S_{i},A_{i})\in \Gamma }log\sum_{L}\exp\left(\phi^{⊤}\cdot \mu \left(A,S,L\right)\right)/Z_{1}-\sum_{k=1}^{K}\frac{1}{2\sigma^{2}}\|\phi_{k}{\|}^{2} \end{array}$$ (6)

where we suppose *ϕ* ∈ **R** ^*K*^ (*K* = *K*_1_+*K*_2_). The objective function 𝓛(Γ;*ϕ*) is the summation of log-likelihood of all training samples minus a regularization term. The second term $\frac{1}{2\sigma^{2}}\|\phi {\|}^{2}$ is a an *L*_2_-norm regularization when parameters are assumed to be Gaussian distributed with variance *σ* ^2^. It is set to avoid overfitting.

To achieve the optimal parameter ϕ*, we can take a partial derivative of 𝓛(Γ;*ϕ*) with respect to each entity *ϕ*_*k*_ as:

$$\begin{array}{c} \frac{\partial L(\Gamma ;\phi )}{\partial \phi_{k}}=\sum_{(S_{i},A_{i})\in \Gamma }\frac{\partial log\sum_{L}\exp\left(\phi^{⊤}\cdot \mu \left(A,S,L\right)\right)/Z_{1}}{\partial \phi_{k}}-\frac{\phi_{k}}{\sigma^{2}} \end{array}$$ (7)

Since *ϕ*_1, *k*_ only exists in the first-order attribute term, the partial derivative of the core of Eq 7 can be derived as:

$$\begin{array}{c} \begin{array}{cc} \frac{\partial log\sum_{L}\exp\left(\phi^{⊤}\cdot \mu \left(A,S,L\right)\right)/Z_{1}}{\partial \phi_{1,k}}= & \frac{\partial log\sum_{L}\exp\left(\phi^{⊤}\cdot \mu \left(A,S,L\right)\right)}{\partial \phi_{1,k}}-\\ & \frac{\partial log\sum_{A^{\prime }\in A}\sum_{L}\exp\left(\phi^{⊤}\cdot \mu \left(A^{\prime },S,L\right)\right)}{\partial \phi_{1,k}}\\ = & \sum_{L}\sum_{p=1}^{P}\sum_{i=1}^{I}P\left(L|S_{i},A_{i};\phi \right)\cdot F_{1,k}\left(l_{i}^{p},s_{i}^{p}\right)-\\ & \sum_{A^{\prime }}\sum_{L}\sum_{p=1}^{P}\sum_{i=1}^{I}P\left(L,A^{\prime }|S_{i};\phi \right)\cdot F_{1,k}\left(l_{i}^{p},s_{i}^{p}\right)\\ = & p_{1,k}\left(A,L,S;\phi \right) \end{array} \end{array}$$ (8)

In the same way, *ϕ*_2, *k*_ only exists in the second attribute term and the partial derivative of the core of Eq 7 is:

$$\begin{array}{c} \begin{array}{cc} \frac{\partial log\sum_{L}\exp\left(\phi^{⊤}\cdot \mu \left(A,S,L\right)\right)/Z_{1}}{\partial \phi_{2,k}}= & \sum_{L}\sum_{p=1}^{P}\sum_{i=1}^{I}P\left(L|S_{i},A_{i};\phi \right)\cdot F_{2,k}\left(l_{i}^{p},l_{i+1}^{p}\right)-\\ & \sum_{A^{\prime }}\sum_{L}\sum_{p=1}^{P}\sum_{i=1}^{I}P\left(L,A^{\prime }|S_{i};\phi \right)\cdot F_{2,k}\left(l_{i}^{p},l_{i+1}^{p}\right)\\ = & p_{2,k}\left(A,L,S;\phi \right) \end{array} \end{array}$$ (9)

With the partial derivative of Eq 7 with respect to both *ϕ*_1, *k*_ and *ϕ*_2, *k*_ above, the parameters can be updated as follows:

$$\begin{array}{c} \begin{array}{cc} \phi_{1,k}^{t}= & \phi_{1,k}^{t-1}+\gamma \frac{\partial L(\Gamma ,\phi )}{\partial \phi_{1,k}}{|}_{\phi =\phi^{t-1}}\\ = & \phi_{1,k}^{t-1}+\gamma \left(\sum_{(S_{i},A_{i})\in \Gamma }p_{1,k}\left(A,L,S;\phi \right)-\frac{\phi_{1,k}^{t-1}}{\sigma^{2}}\right) \end{array} \end{array}$$ (10)

$$\begin{array}{c} \begin{array}{cc} \phi_{2,k}^{t}= & \phi_{2,k}^{t-1}+\gamma \frac{\partial L(\Gamma ,\phi )}{\partial \phi_{2,k}}{|}_{\phi =\phi^{t-1}}\\ = & \phi_{2,k}^{t-1}+\gamma \left(\sum_{(S_{i},A_{i})\in \Gamma }p_{2,k}\left(A,L,S;\phi \right)-\frac{\phi_{2,k}^{t-1}}{\sigma^{2}}\right) \end{array} \end{array}$$ (11)

where *t* means the iteration index; *γ* means the learning rate.

The parameter *ψ* of the inter-sequence sub-model *P*_2_(*A*∣*S*, *ψ*) can be optimized in the same way and we omit the related derivation. For model inference, the optimal sequence label A* can be predicted with the belief propagation algorithm [33]:

$$\begin{array}{c} A^{*}=\arg\max_{A\in A}P\left(A|S;\Theta^{*}\right) \end{array}$$ (12)

where Θ* = {ϕ*, ψ*}.

## Experiment Method

### Data

The proposed method is evaluated on the popular datasets in both RGB and depth modalities, including KTH, TJU, MSR Daily Activity 3D (MDA) respectively shown in Fig 2a, 2b and 2c. We will briefly introduce the datasets as follows.

- **KTH**: KTH [22] contains 6 action categories: 1. boxing, 2. hand clapping, 3. hand waving, 4. jogging, 5. running and 6. walking. Each action was performed by 25 people in 4 different scenarios, including indoor, outdoor, changes in clothing and variations in scale. Each video sample contains one subject engaged in a single activity in a certain condition. For fair comparison, we strictly followed the experiment setting as [34] in the “split” manner.
- **MDA**: MSR Daily Activity 3D [35] is very challenging since it captures the human daily activities with human-object interaction. It consists of 16 action(1.drink, 2.eat, 3.read book, 4.call cell phone, 5.write on a paper, 6.use laptop, 7.toss paper, 8.play game, 9.play guitar, 10.use vacuum cleaner, 11.cheer up, 12.sit still, 13.lay down on sofa, 14.walk, 15.stand up, 16.sit down). Each person performs one action in two scenarios, sitting on sofa and standing. We eliminated 4 foot joint positions and only used the other 16 key joints for partwise BoW generation because the foot regions are usually out of the range of Kinect. The Leave-One-Subject-Out strategy is leveraged for evaluation.
- **TJU**: We contributed a public dataset, TJU, to provide an action dataset with more samples and action categories. TJU was recorded in the front view and RGB image(640 × 480), depth image(640 × 480) and skeleton were recorded. The dataset contains 22 action categories: 1. walking, 2. jogging, 3. running, 4. boxing, 5. waving, 6. clapping, 7. bend, 8. jacks, 9.jump, 10. p-jump, 11. side, 12. single-wave, 13. draw-x, 14. draw-tick, 15. draw-circle, 16. kick, 17. side-kick, 18. tennis-swing, 19. tennis-serve, 20. side-box, 21. p-bend and 22. sit-down. Each of the 22 actions was performed four times by 20 people. There are totally 1760 samples. The dataset is splitted into two parts, the first 12 subjects for training and the rest for test. TJU is challenging since it contains the more complex action samples with high intra variation and was captured in both light and dark environments.

### Implementation Details

For RGB datasets (KTH and TJU), we utilized STIP [36] for spatiotemporal interest point localization and representation because it has shown superior performances in the BoW+SVM framework [34]. For depth dataset (MDA), the local HON4D descriptor and the 3Djoint position feature were utilized. As Oreifej [37], we concatenated HON4D and 3Djoint position features for representation and further utilized them for dictionary learning and BoW representation. The original parameter settings were implemented to extract STIP and HON4D features for fair comparison. For KTH, the part-based model [17] was implemented on each frame to localize body part regions. For TJU and MDA, we directly used the skeleton data provided in each dataset for body part localization. Then the space-time feature points were grouped into different body parts in terms of the geometric distances between the point and the center of each part region. To construct the visual vocabulary of individual part, we clustered 20,000 feature points of each part region sampled from the training videos with the K-means algorithm. The number of visual words was empirically set with 100, which showed satisfactory performance for partwise ST-AUS representation. The ST-AUS in our experiment lasted for 30 frames in temporal domain and the overlap between two adjacent ST-AUSs was set with 15.

## Experimental Results

To show the superiority of the proposed method, the following two comparison experiments were implemented.

### Comparison against other fusion methods

**Fig 4:**
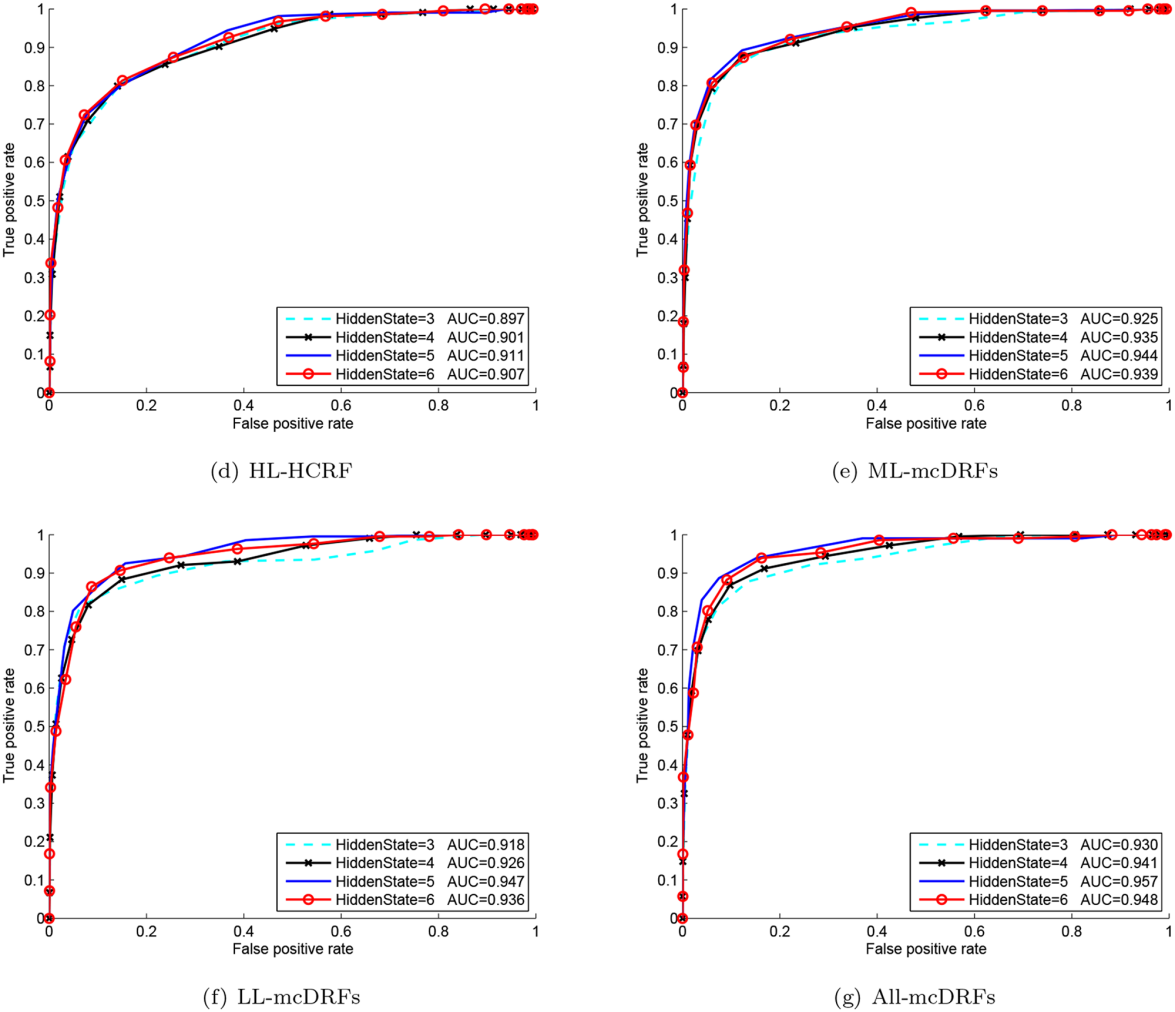
ROC and AUC of KTH with different hidden states.

**Fig 5:**
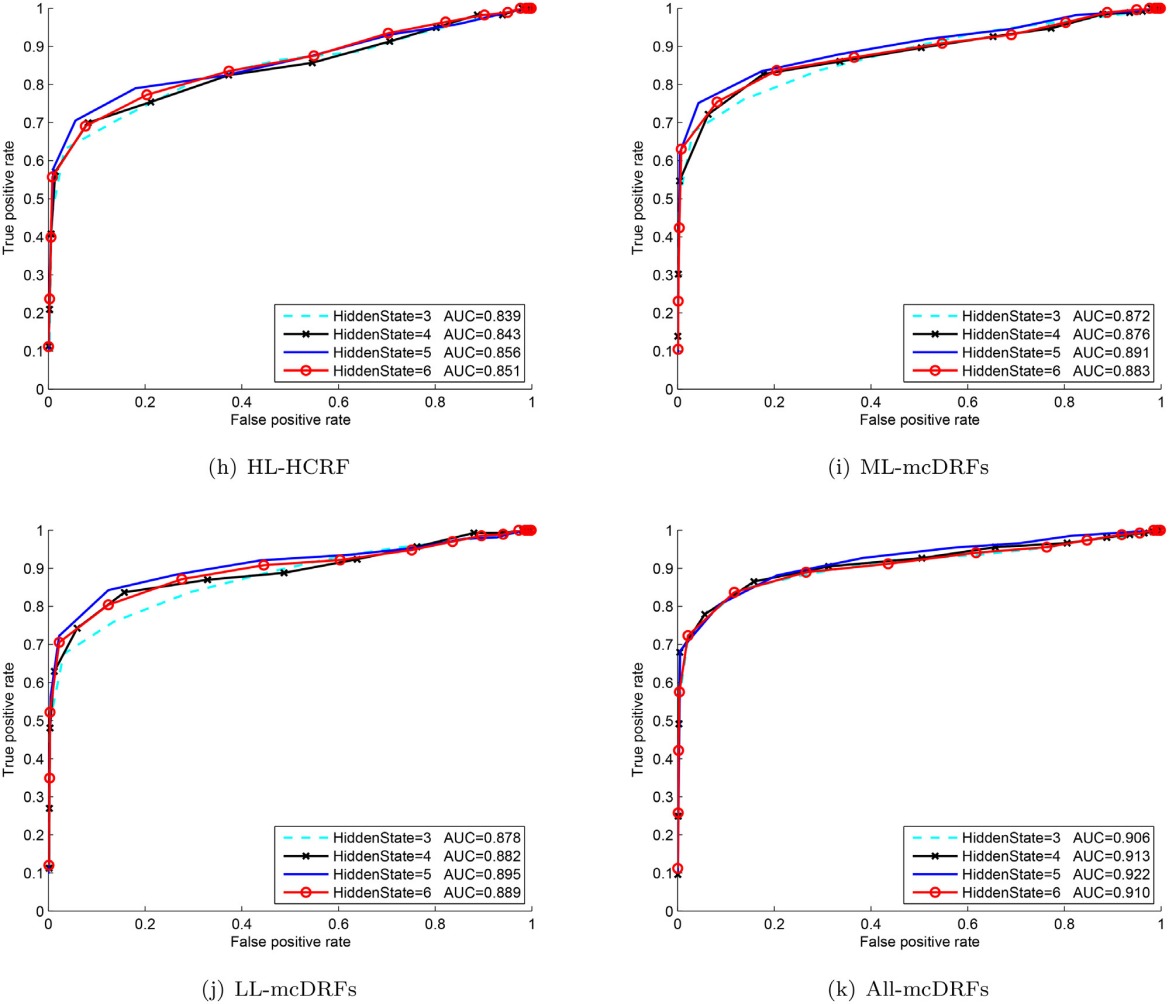
ROC and AUC of TJU with different hidden states.

**Fig 6:**
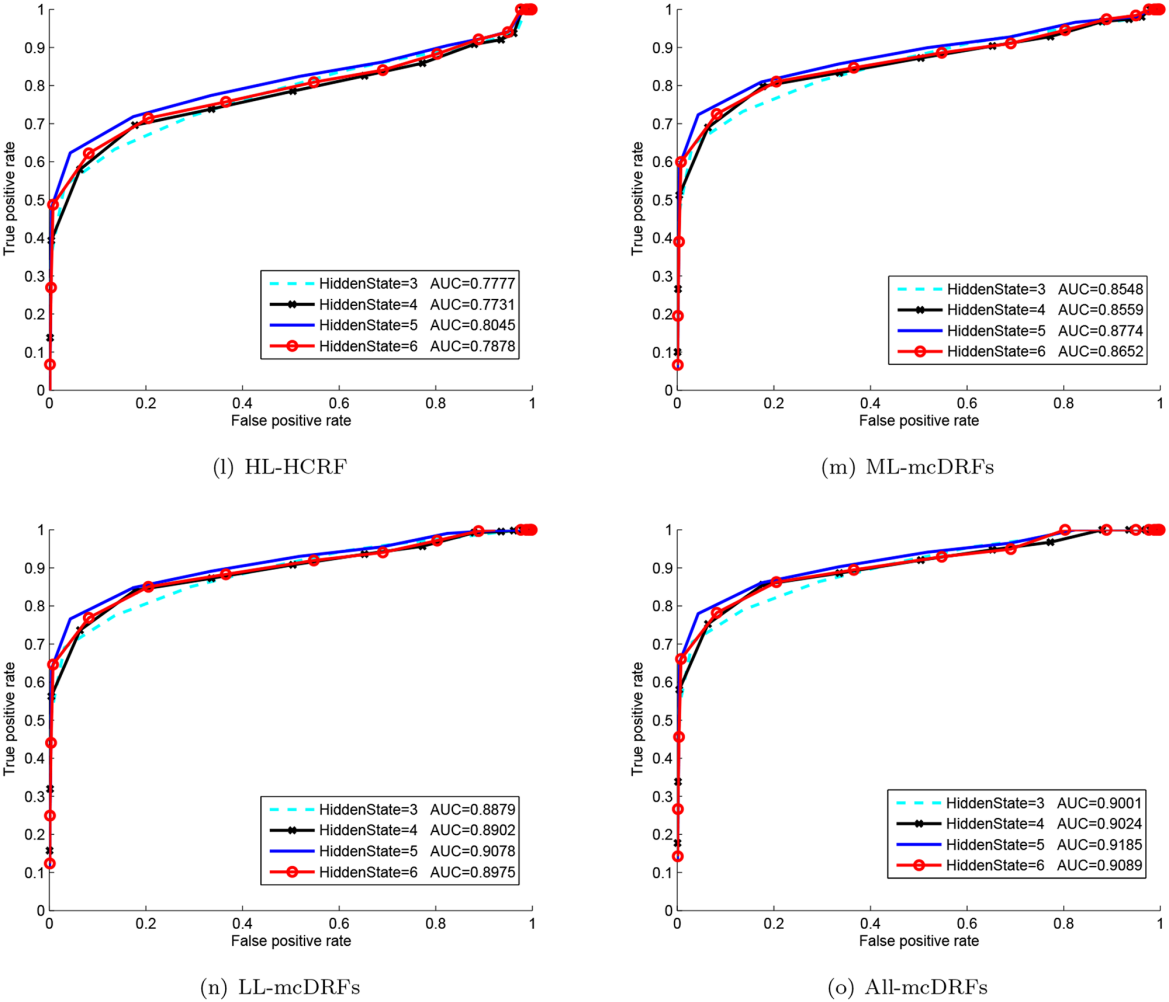
ROC and AUC of MDA with different hidden states.

With the ST-AUS representation, the proposed MTCRFs model can be trained and utilized for action recognition based on the Maximum A Posteriori criteria. To select the best hidden state for temporal modeling, we plotted the ROC curve of each hidden state number and the best parameter can be selected when the area under curve (AUC) of the corresponding ROC reached the maximum. In our experiments, we varied the number of hidden states from 3 to 6 per chain for parameter selection. From Figs 4, 5 and 6, it is obvious that the MTCRFs model on each dataset can achieve the best performance with *hidden*_*state* = 5.

To show the proposed method can benefit sequential dynamic learning, we implemented the following strategies for comparison:

- HL-HCRF: HCRF model trained only with HL-AUS (as shown in Fig 1(h)), which utilized the bodywise feature for temporal modeling.
- LL-DDF: Direct decision-level fusion by linearly combining the posterior probabilities of HCRF models, trained with LL-AUS1, LL-AUS2, LL-AUS3, LL-AUS4 respectively, with equal weights.
- ML-DDF: Direct decision-level fusion by linearly combining the posterior probabilities of HCRF models, trained with ML-AUS1 and ML-AUS2 respectively, with equal weights.
- All-DDF: Direct decision-level fusion by linearly combining the posterior probabilities of HCRF models, trained with 7 components of ST-AUS respectively, with equal weights.
- LL-DFF: HCRF model trained with direct feature-level fusion of LL-AUS1, LL-AUS2, LL-AUS3, LL-AUS4.
- ML-DFF: HCRF model trained with direct feature-level fusion of ML-AUS1 and ML-AUS2.
- All-DFF: HCRF model trained with direct feature-level fusion of 7 components of ST-AUS.
- LL-MTCRFs: MTCRFs model trained with LL-AUS1, LL-AUS2, LL-AUS3, LL-AUS4 as sequential observations.
- ML-MTCRFs: MTCRFs model trained with ML-AUS1 and ML-AUS2 as sequential observations.
- All-MTCRFs: MTCRFs model trained with 7 components of ST-AUS as sequential observations.

**Fig 7:**
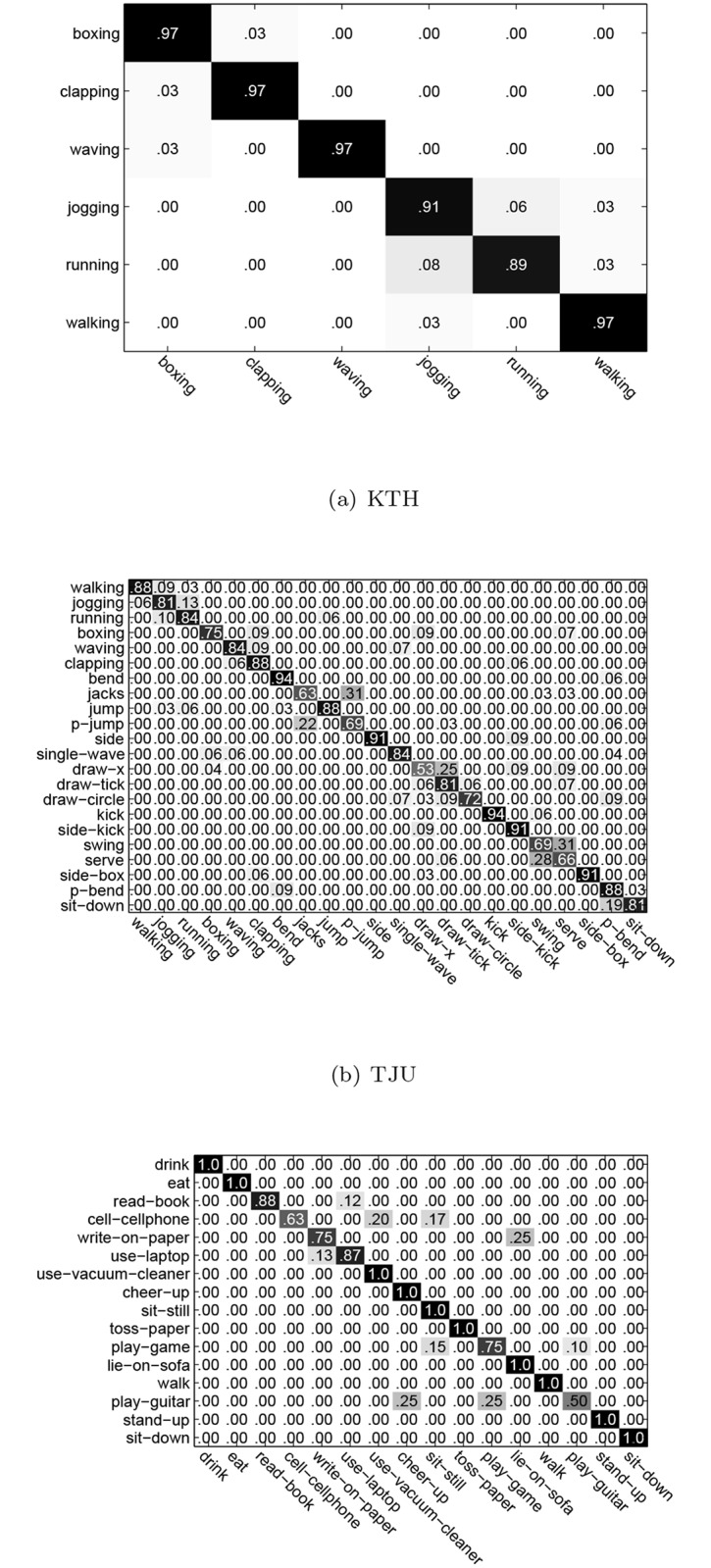
The confusion matrixes of All-MTCRFs on KTH, TJU, and MDA.

The comparison results are shown in Table 1. The confusion matrixes of the optimal performance on three datasets by All-MTCRFs are respectively shown in Fig 7a, 7b and 7c.

**Table 1:** Performance Comparison on KTH, TJU, and MDA (%).

Method	KTH	TJU	MDA
HL-HCRF	90.0	80.5	71.2
ML-DDF	91.0	81.4	75.7
LL-DDF	91.4	82.6	77.4
All-DDF	91.9	84.3	78.1
ML-DFF	91.4	84.7	81.6
LL-DFF	91.9	85.2	81.9
All-DFF	92.4	86.8	84.4
ML-MTCRFs	92.9	86.2	86.7
LL-MTCRFs	93.3	88.1	88.0
All-MTCRFs	**94.8**	**90.5**	**89.8**

From Table 1 we can see that HL-HCRF worked in a traditional manner for HCRF learning simply with global feature and only acieved 90.0%, 80.5% and 71.2% for KTH, TJU, and MDA respectively. ML-DDF, LL-DDF, and All-DDF worked in a decision-fusion manner and cannot take advantage of the temporal context of all sequences for sequential structure learning. It can only improve the performance slightly. Comparatively, ML-DFF, LL-DFF, and All-DFF worked in a feature-fusion manner and can leverage the temporal context of all partwise sequences for sequential structure learning. Consequently the feature-level fusion method can further augment the performance. Since the proposed MTCRFs can preserve and learn the temporal context of individual ST-AUS while relaxing the interaction inbetween to handle the asynchronization, it can achieve the best accuracy of 94.8%, 90.5% and 89.8% on three datasets respectively and consistently outperform all the others.

**Fig 8:**
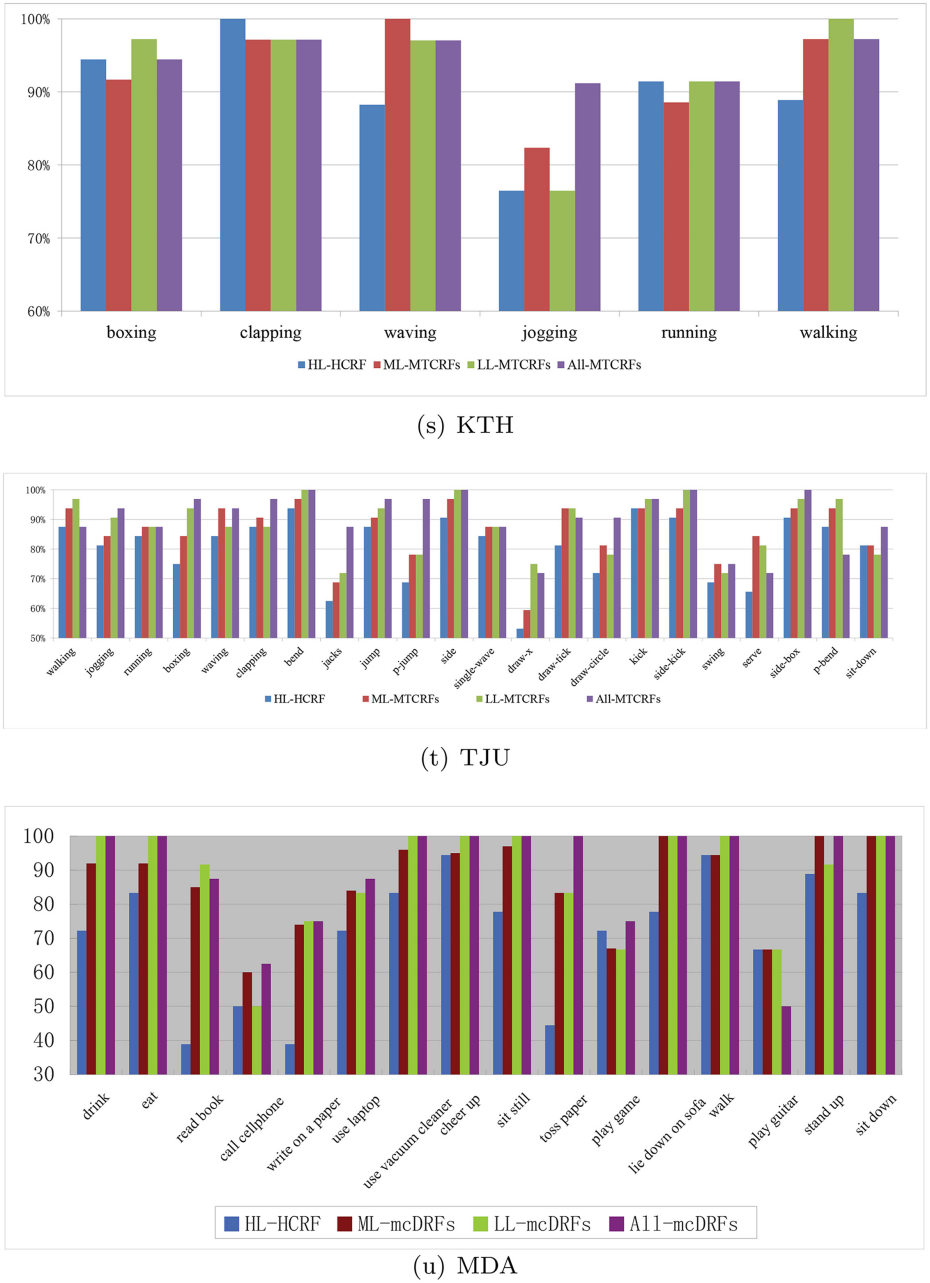
Action category-wise comparison among HL-HCRF, LL-MTCRFs, ML-MTCRFs, and All-MTCRFs on KTH, TJU, and MDA.

The action category-wise comparison among HL-HCRF, LL-MTCRFs, ML-MTCRFs, and All-MTCRFs for three datasets are listed in Fig 8a, 8b and 8c. For KTH, All-MTCRFs can work better or equally to the others in 4 out of 6 actions. Especially it can drastically improve the accuracy of Jogging from around 80% to 91.2%, which is the most challenging one in KTH. For TJU, All-MTCRFs can rank 1st in 17 out of 22 action. It can augment the performance of 4 actions (clapping, jacks, p-jump, draw-circle) with more than 10% accuracy. For MDA in the depth modality, All-MTCRFs can also improve the performance and rank 1st in 14 out of 16 action. It can augment the performance of 6 actions (drink, read book, write on a paper, sit still, toss paper, lie down on sofa) with more than 20% accuracy. This comparison demonstrates that the proposed method is more robust to high intra variation caused by more complex actions and diverse environments. The action-wise comparison among the first three methods in Fig 8 shows that LL-MTCRFs can work better or equally to HL-HCRF and ML-MTCRFs in 3 out of 6 actions in KTH, 16 out of 22 actions in TJU, 12 out of 16 actions in MDA. It further demonstrates that the body part-induced ST-AUS representation is more discriminative for local dynamic description and consequently facilitates human action recognition.

### Comparison against state-of-the-art methods

To show the superiority of the proposed method, we compared its performances on KTH, TJU, and MDA to the state-of-the-art methods. Tables 2 and 3 show the comparison on KTH & MDA. The performances of the competing methods are cited from the corresponding papers. From Tables 2 and 3 it is obvious that the proposed method can outperform the competing methods in both RGB and depth modalities. Especially, the proposed method can outperform the slow feature-based method [47], which can also explore the temporal context. Theoretically, Zhang *et al*. [47] utilized the slow feature learning for frame-wise feature transformation and further computed the accumulated squared derivative (ASD) feature of video-wise representation by leveraging the statistical characteristics of frame-wise slow features. Although the ASD feature also conveys the temporal characteristics, it loses the multiple sequential context represented by the proposed part-induced spatiotemporal action unit sequence. Therefore, it is reasonable that the proposed method can outperform the slow feature-based method.

**Table 2:** Comparison with state of the arts on KTH.

Method	Year	Accuracy(%)
Fathi *et al*.	(CVPR2008) [38]	90.5
Niebles *et al*.	(IJCV2008) [39]	83.3
Laptev *et al*.	(CVPR2008) [28]	91.8
Kläser *et al*.	(BMVC2008) [3]	91.4
Wang *et al*.	(BMVC2009) [34]	92.1
Gilbert *et al*.	(ICCV2009) [40]	94.5
Taylor *et al*.	(ECCV2010) [41]	90.0
Kovashka *et al*.	(CVPR2010) [42]	94.5
Le *et al*.	(CVPR2011) [43]	93.9
Wang *et al*.	(CVPR2011) [44]	93.8
Minhas *et al*.	(TCSVT2012) [45]	94.4
Ballan *et al*.	(TMM2012) [46]	92.7
U-SFA	(TPAMI2012) [47]	84.7
S-SFA	(TPAMI2012) [47]	88.8
D-SFA	(TPAMI2012) [47]	91.2
SD-SFA	(TPAMI2012) [47]	93.5
Ji *et al*.	(TPAMI2013) [48]	90.2
Ma *et al*.	(TCSVT2013) [49]	94.4
Proposed		**94.8**

**Table 3:** Comparison with state of the arts on MDA.

Method	Year	Accuracy(%)
HOG	CVPR2008 [28]	79.1
LOP	CVPR2012 [50]	42.5
Joint Position	CVPR2012 [50]	68.0
Actionlet Ensemble	CVPR2012 [50]	85.8
DCSF	CVPR2013 [51]	83.6
DCFS+Joint	CVPR2013 [51]	88.2
Proposed		**90.5**

Table 4 shows the comparison on TJU. Since TJU is a new dataset prepared by our group and no off-the-shelf comparison available, we re-implemented six representative methods for comparison: 1) STIP-BoW+SVM: the standard BoW+SVM method with the STIP feature [28]; 2) 3DHOG-BoW+SVM: the standard BoW+SVM method with the 3DHOG feature [3]; 3) BoW+SRC: the standard BoW representation with STIP and sparse representation-based classification [52]; 4) BoW+CRC: the standard BoW representation with STIP and collaborative representation-based classification [53]; 5) HL-AUS+HMM: bodywise action unit sequence with STIP and hidden markov model [54]; 6) HL-AUS+semi-DRF: bodywise action unit sequence with STIP and semi-Markov discriminative random fields [55]. The first four methods belong to the popular BoW-based methods for classification without utilizing temporal context. Therefore it is expected that their performances are relatively lower than the other temporal inference methods. HMM and semi-DRF are two popular models which leverage sequence structure for temporal modeling. HMM is theoretically limited by its inability to accommodate long-range dependencies among observations or multiple overlapping features because they assume that the observations are conditionally independent. Therefore semi-DRF works better than HMM. However, both HMM and semi-DRF cannot take advantage of multiple partwise ST-AUS simultaneously for sequence structuring learning and the latent correlation discovery. Consequently, the proposed MTCRFs model can achieve the best performance of 90.5% accuracy.

**Table 4:** Comparison with state of the arts on TJU.

Method	Accuracy(%)
STIP-BoW+SVM	83.3
3DHOG-BoW+SVM	82.6
BoW+SRC	83.9
BoW+CRC	84.6
HL-AUS+HMM	85.4
HL-AUS+semi-DRF	87.0
Proposed	**90.5**

## Conclusion

In this paper we propose a novel human action recognition method based on multi-task conditional random fields model. For feature representation, we propose the part-induced spatiotemporal action unit sequence to represent each action sample with multiple partwise sequential feature subspaces. For model learning, we propose the multi-task conditional random fields (MTCRFs) model to discover the sequence-specific structure and the sequence-shared relationship. Specifically, we propose the probabilistic model and the corresponding learning and inference methods to discover temporal context within individual action unit sequence and the latent correlation among different body parts. The comparison experiments demonstrated its superiority on two RGB human action datasets, KTH & TJU, and one depth dataset, MSR Daily Activity 3D.
